# Cryo-EM structures of *Acinetobacter baumannii* glycerophospholipid transporter

**DOI:** 10.1038/s41421-020-00230-5

**Published:** 2020-11-19

**Authors:** Yuanyuan Zhang, Qiongxuan Fan, Ximin Chi, Qiang Zhou, Yanyan Li

**Affiliations:** 1 Center for Infectious Disease Research, Westlake Laboratory of Life Sciences and Biomedicine, Westlake University, Hangzhou, Zhejiang 310024 China; 2 Key Laboratory of Structural Biology of Zhejiang Province, Westlake University, Hangzhou, Zhejiang 310024 China; 3 Institute of Biology, Westlake Institute for Advanced Study, Hangzhou, Zhejiang 310024 China

**Keywords:** Structural biology, Membrane trafficking

Dear Editor,

*Acinetobacter baumannii* is a prevalent nosocomial pathogen that causes serious threat in health care institutions^1^. *A. baumannii* has demonstrated resistance to a wide array of antibiotics, including the last-resort colistin or polymyxin B^2–4^. The MlaFEDB-mediated glycerophospholipid (PL) transport was reported to play an important role in maintaining the integrity of the lipid membrane^2,3^. The MlaFEDB complex is an ATP-binding cassette transporter to actively translocate the phospholipids between the inner membrane and the periplasmic protein MlaC^4,5^. Despite the progress that has been made in understanding the function of the MlaFEDB complex by genetic and biochemical strategies in *A. baumannii*, questions still remain open surrounding the directionality in glycerophospholipids transport via MlaFEDB in both retrograde and anterograde transport^6,7^. The low-resolution structure of the MlaFEDB complex from *A. baumannii* at 8.7 Å has provided initial structural insights into this complex^8^. However, the molecular details of the transport complex assembly, the interactions with glycerophospholipids, and the transport mechanisms of the MlaFEDB in *A. baumannii* remain enigmatic.

To chase the high-resolution structure of MlaFEDB, we overexpressed the *A. baumannii mla*FEDCB operon in *Escherichia coli* BL21(DE3) pLyS. The MlaFEDB proteins formed a stable complex and were reconstituted into lipid nanodisc (Fig. 1a and Supplementary Fig. S1a–c). The ATPase activity of *A. baumannii* MlaFEDB is approximately three times that of the *E. coli* MlaFEDB observed in our previous study^9^ (Fig. 1b, left). The ATPase activity exhibits the *K*_m_ around 0.085 ± 0.005 mM and the *V*_max_ around 373.7 ± 4.08 mol ATP per min per mol MlaFEDB (Fig. 1b, right), suggesting a high ATP-binding affinity during the transportation cycle.

**Fig. 1:**
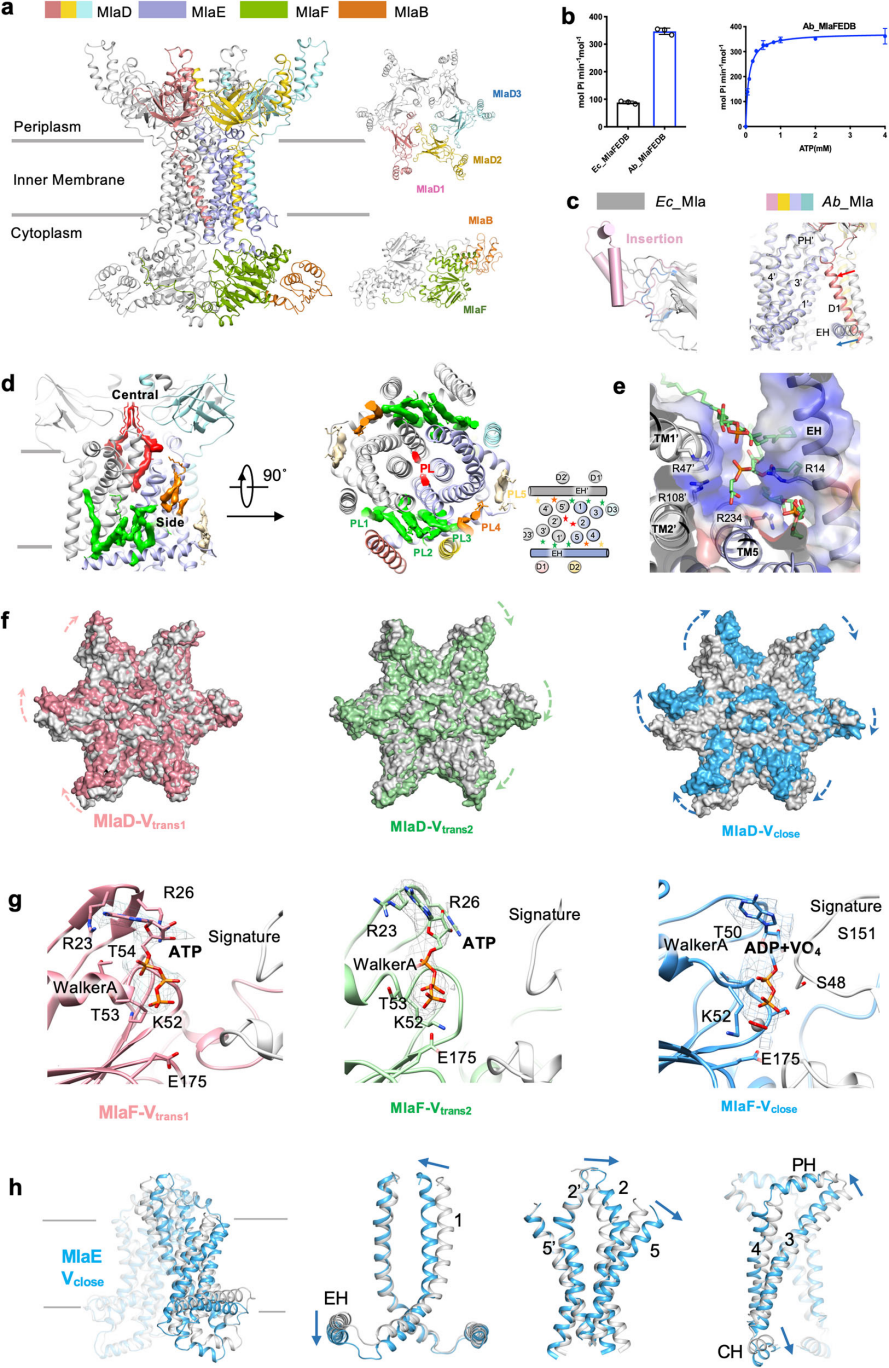
Cryo-EM structures of glycerophospholipid transporter complex MlaFEDB from *A. baumannii*. **a** Cryo-EM structure of the MlaFEDB complex. MlaD, MlaE, MlaF and MlaB are colored separately. **b** The ATPase activities of MlaFEDB from *A. baumannii* and *E. coli*. Each point represents mean ± SD. **c** Overlay of the MCE domain of MlaD (left) and TMs of MlaED between *A. baumannii* (color) and *E. coli* (gray, PDB 7CGE). **d** Cross-sectional view (left) and periplasmic view (middle) of the model with phospholipid binding. Twelve glycerophospholipids (PLs) are modeled and are shown as stars in the cartoon view (right). Two PLs bound in the central cavity are colored red. PL1–3, PL4, and PL5 bound in one side cavity are shown and colored green, orange, and yellow, respectively. **e** Electrostatic surface view of the interaction between PL1–3 and MlaE, indicating areas of positive charge (blue). **f** Surface overlay of the MCE domain of MlaD between the nucleotide-free (gray) and V_trans1_, V_trans2_, and V_close_ conformations. The arrows indicate the movement of MCE. **g** Cryo-EM densities for the nucleotides bound at the ATP-binding sites of two subunits of MlaF in V_trans1_ (raspberry), V_trans2_ (green), and V_close_ (blue). ATP or ADP-VO4 binds to the Walker A and Signature motifs. R23, R26, K52, T53, T54, and E175 participate in the binding. **h** Structure overlay of MlaE in the nucleotide-free (gray) and V_close_ (blue) conformations. Blue arrow indicates the movement of TMD of MlaE.

The structure of MlaFEDB complex in nanodisc in nucleotide-free form was determined by cryogenic electron microscopy (cryo-EM). The two-dimensional class averages of MlaFEDB showed clear structural features (Supplementary Fig. S2a). In our previous studies, *E*. *coli* MlaFEDB exhibited twofold symmetry, with bound glycerophospholipid densities along the axis of symmetry^9^ (PDB 7CGE). To improve and confirm densities along the central axis, no symmetry was applied during the reconstruction of *A. baumannii* MlaFEDB (Fig. 1a and Supplementary Fig. S2b). The final map of the MlaFEDB complex was well defined at 3.1 Å resolution, allowing us to build a nearly complete model for MlaFEDB, which includes a high-resolution structure of transmembrane helices (TMs) of MlaE and MlaD (Supplementary Fig. S3 and Table S1). Consistent with the previous reported low-resolution *A. baumannii* MlaFEDB map^8^ and our *E. coli* MlaFEDB map^9^ (EMD-30355), the MlaF (cytoplasmic nucleotide-binding domain, NBD), MlaE (transmembrane domain), MlaD (periplasmic phospholipid-binding domain), and MlaB (cytoplasmic auxiliary domain) subunits are assembled in a stoichiometry of 2:2:6:2 (Fig. 1a).

On the periplasmic side, the mammalian cell entry (MCE) domain of MlaD is assembled as a hexameric ring (Fig. 1a). The MlaD of *A. baumannii* shares 27.2% identity with the MlaD of *E. coli*. The MCE domain is composed of a seven-stranded β-barrel fold. Extra density corresponds to a 47 amino acid α-helices insert between the fourth and fifth strand β-sheet (Fig. 1c left and Supplementary Fig. S4). The function of the insertion is unclear. On the cytoplasmic side, two subunits of MlaF interact with two subunits of MlaB (Supplementary Fig. S5a) and the C terminus of one MlaF subunit extends to wrap around the opposite subunit of MlaF and the neighboring MlaB, forming close contacts (Fig. 1a and Supplementary Fig. S5b).

In the transmembrane region, the MlaFEDB complex consists of a total of 18 long helices in the inner membrane, formed by 2 subunits of MlaE and 6 subunits of MlaD. Three protomers of the MlaD hexamer (MlaD1, MlaD2, and MlaD3) incorporate with one subunit of MlaE (Fig. 1a and Supplementary Fig. S6a). Two subunits of MlaE form a homodimer and each subunit contains one elbow helix (EH), five TMs, and one coupling helix and one periplasmic helix (Supplementary Fig. S6a, right). The EH is amphipathic and runs parallel to the inner membrane representing the novel feature of MlaFEDB complex. The TM of MlaD1 (D1) and MlaD2 (D2) interact tightly with EH of MlaE (Supplementary Fig. S6b, c). The TM of MlaD3 (D3) interacts with TM1 and TM3 (Supplementary Fig. S6a). The assembly of TM1–5 in MlaE are essentially identical to that in *E. coli* MlaE^9^ (PDB 7CGE). Notably, the EH moves inward to the TMs of MlaE coordinating inward movement of D1 and D2, and forming a smaller side cavity in comparison to the cavity formed by MlaE and MlaD in *E. coli*^9^ (Fig. 1c right and Supplementary Fig. S6d).

Cryo-EM structure of MlaFEDB in nucleotide-free state also reveals glycerophospholipids bound inside the MlaE and MlaD, suggesting that the overexpressed *A. baumannii* MlaFEDB can bind glycerophospholipids from *E. coli*. Clusters of diacyl glycerophospholipid densities were resolved to be located in the central cavity and side cavities symmetrically (Fig. 1d). Two glycerophospholipids (PL center) are bound in the central cavity (Fig. 1d). The well-defined densities are resolved with one acyl chain reaching down into the MlaE hydrophobic cavity and the other reaching upwards into the cavity formed by MCE of MlaD (Supplementary Fig. S7a, d), which is consistent with the lipids found in *E. coli* (PDB 7CGE). Glycerophospholipid densities are also found on the two side cavities formed by MlaE and MlaD (Fig. 1d). Five diacyl glycerophospholipids (PL1–5) are built in one side cavity (Fig. 1d, right), which is formed by EH, TM3’ TM1’, TM5, and TM4 from MlaE and D3’, D1, and D2 from MlaD. The head groups of PL1–3 are pointing down and are accommodated by a cluster of positively charged residues from MlaD, which include Arg47, Arg108, Arg14, and Arg234 (Fig. 1e and Supplementary Fig. S7d, e). PL4 is trapped in a position close to the outer leaflet of the inner membrane and PL5 locates close to the end of EH (Fig. 1d and Supplementary Fig. S7c). Extra trace of glycerophospholipid’s tail densities exist in the area close to the PL5. The bound glycerophospholipids in the side cavity may play a role in the regulation of glycerophospholipid translocation or structural integrity of the complex.

To understand the mechanism of glycerophospholipid translocation coupled with the conformational change, distinct conformations of MlaFEDB were trapped using sodium *ortho*-vanadate^10^. Vanadate acts as a potent inhibitor of ATPase by forming an ADP–vanadate complex that mimics the intermediate state formed during hydrolysis. In the presence of 1 mM vanadate, the ATPase activity of MlaFEDB is reduced by ~50% (Supplementary Fig. S1d), indicating an incomplete inhibition state. Three individual conformations were classified and refined to 4.0, 3.6, and 4.0 Å, and were named V_trans1_, V_trans2_, and V_close_, respectively (Supplementary Figs. S8,S9 and Table S1). All three structures display bound nucleotides at the ATP-binding sites (Fig. 1g). However, the maps are of insufficient quality to distinguish between ADP, ATP, and ADP–vanadate.

Comparison of V_trans1_ and V_trans2_ to nucleotide-free structure shows the conformational rearrangement of the MlaD. MlaD hexametric ring rotates clockwise at different levels (Fig. 1f and Supplementary Movies S1 and S2), whereas MlaFEB remains unchanged. The structure of V_close_ shows the tight dimerization and close proximity of the NBD of MlaF subunits, caused by the trapping of ADP–vanadate or ATP at the ATP-binding sites comprising the Walker A, Walker B motifs, and the signature motif (Fig. 1g). The dimerization of the NBDs engages the coupled structural rearrangements of MlaE and MlaD, respectively. The MlaD ring rotates clockwise and the two subunits of MlaE squeeze towards each other to collapse the central phospholipid-binding cavity to extrude lipids (Fig. 1f, h and Supplementary Movie S3). The conformational changes from nucleotide-free to V_close_ in *A. baumannii* are consistent with the changes captured from nucleotide-free (PDB 7CGE) to EQ_close_ (PDB 7CH0) in *E. coli*^9^. No bound glycerophospholipid densities were observed in the central cavity in V_close_.

In summary, we characterize four structures of the *A. baumannii* MlaFEDB complex in nucleotide-free and vanadate-trapped conformations using single-particle cryo-EM. The 3.1 Å resolution structure of nucleotide-free MlaFEDB reveals molecular details of the whole complex assembly and the glycerophospholipid binding, suggesting the unique and conserved features as compared to the *E. coli* MlaFEDB. The three vanadate-trapped structures of MlaFEDB reveal different levels of conformational rearrangement of MlaD and MlaE coupled with nucleotide binding and glycerophospholipid translocation. Our findings uncover the binding sites and potential pathway of glycerophospholipid translocation and provide structural insights into the glycerophospholipid transport mechanism mediated by MlaFEDB in *A. baumannii*.

## Supplementary information

Supplemental information

Supplementary Video S1

Supplementary Video S2

Supplementary Video S3

## Data Availability

Four three-dimensional cryo-EM density maps of *A. baumannii* MlaFEDB in nanodiscs have been deposited in the Electron Microscopy Data Bank under accession numbers EMD-30525 (nucleotide-free state of MlaFEDB), EMD-30526 (V_trans1_ state of MlaFEDB), EMD-30527 (V_trans2_ state of MlaFEDB), and EMD-30528 (V_close_ state of MlaFEDB). Four atomic coordinates for the atomic models have been deposited in the Protein Data Bank under accession numbers 7D06 (nucleotide-free state of MlaFEDB), 7D08 (V_trans1_ state of MlaFEDB), 7D09 (V_trans2_ state of MlaFEDB), and 7D0A (V_close_ state of MlaFEDB).

## Supplementary information

**Supplementary Information** accompanies the paper at (10.1038/s41421-020-00230-5).
