# Supplementary material for: Cryo-EM structures of Acinetobacter baumannii glycerophospholipid transporter
Source: Cell Discov. 2020 Nov 19;6:86. doi: 10.1038/s41421-020-00230-5 (PMC7677376; doi:10.1038/s41421-020-00230-5)
Supplement: Supplementary file 1 — Supplemental information [file 41421_2020_230_MOESM1_ESM.pdf]

Supplementary information

Supplementary figures

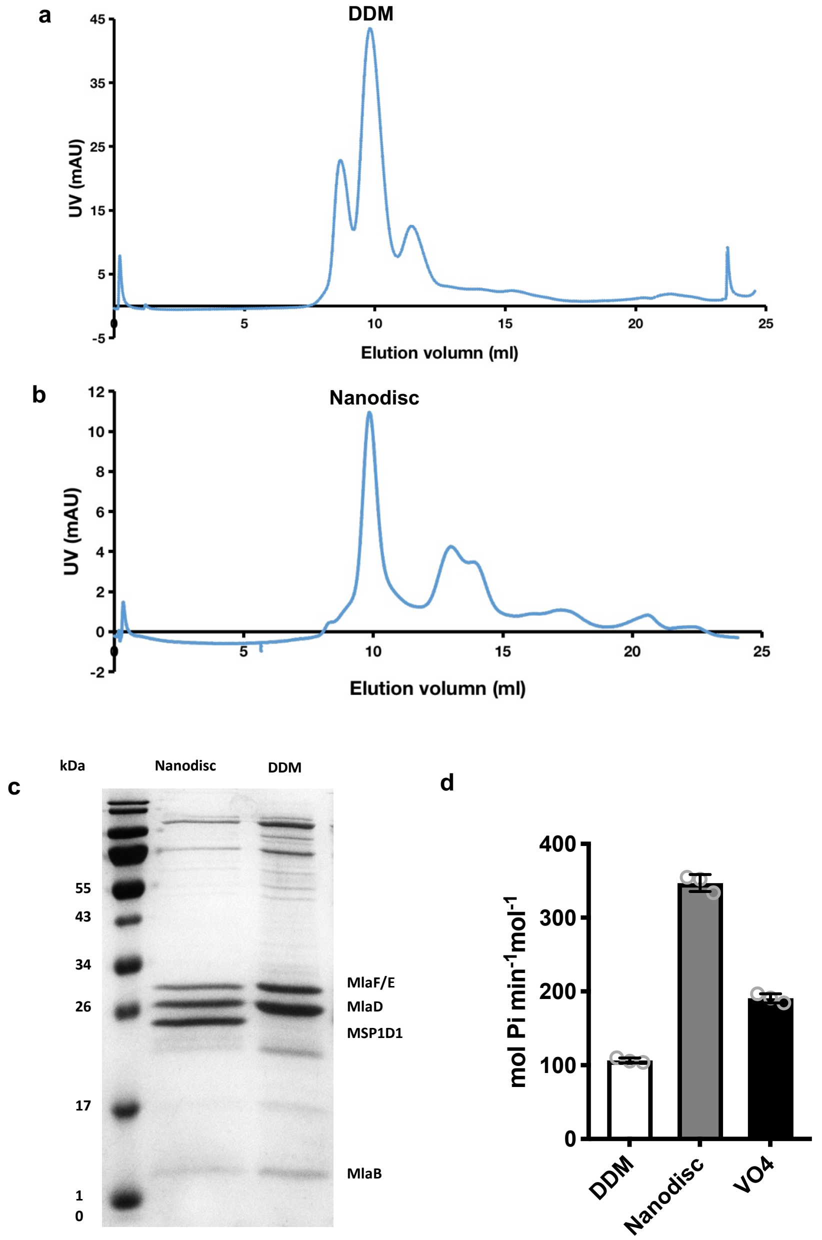

Supplementary Fig. S1 | Purification and functional characterization of MlaFEDB from *A. baumannii.* **a**, Gel-filtration chromatography profile of MlaFEDB in DDM. **b**, Gel-filtration chromatography profile of MlaFEDB in nanodiscs. **c**, SDS-PAGE gel of purified MlaFEDB in DDM and in nanodiscs. **d**, ATPase activity of MlaFEDB in DDM, Nanodisc and in the presence of ATP and 1mM vanadate. Each point represents mean ± s.d. of three separate measurements.

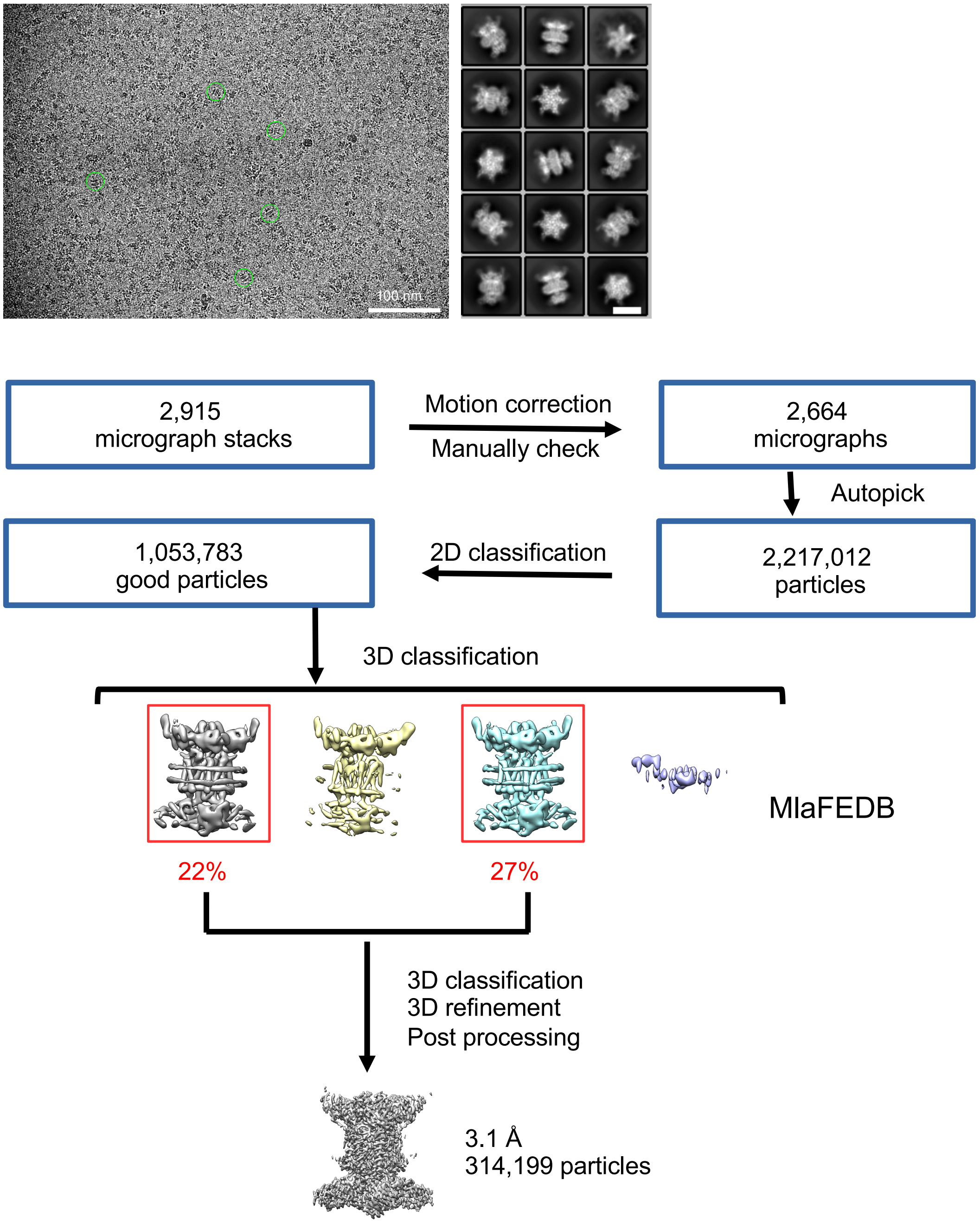

Supplementary Fig. S2 | Image processing for the cryo-EM data of MlaFEDB in nanodiscs. **a,** Representative cryo-EM image and 2D class averages of cryo-EM particle images of nucleotide free MlaFEDB in nanodiscs. **b,** Flow chart for cryo-EM data processing. For details, see ‘Data processing’ in the Methods section.

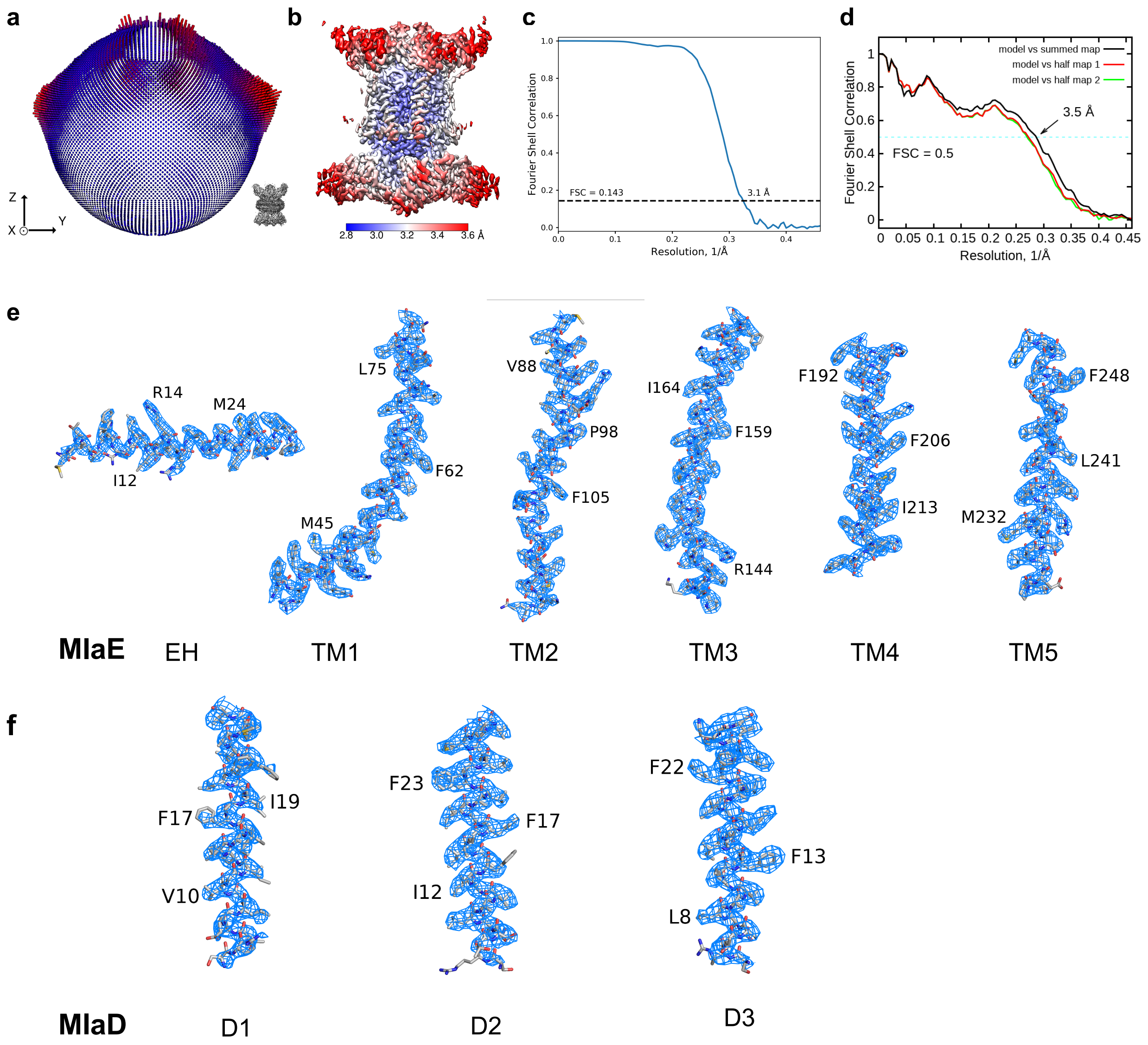

Supplementary Fig. S3 | Single-particle cryo-EM analysis of MlaFEDB in nanodiscs. a-d cryo-EM analysis of MlaFEDB. **a**, Angular distribution of particle images in MlaFEDB. b, Local resolution of the final cryo-EM map of MlaFEDB. **c**, Gold standard FSC curve for the 3D refinement of MlaFEDB. **d**, FSC curve of MlaFEDB between the atomic model and the final map with indicated resolution at FSC=0.5 (black); FSC curve between half map 1 (red) or half map 2 (green) and the atomic model refined against half map 1. **e**, Cryo-EM densities superimposed with the atomic model for individual TM helices in MlaE. **f**, Cryo-EM densities superimposed with the TM helices in MlaD.

a

```
P64604 MLAD_ECOLI          1    MQTKKNEIWVGIFLLAALLAALFVCLKAANVTSIRTEPTYTLYATFDNIGGLKARSPVSI      60
V5V921 V5V921_ACIBA        1    MKSRTSELAVGIFVIIFGIALFFLAMKVSGLVGTNLSDGYTMKAQFDNVNGLKPRAKVTM      60
                                *::::..*: ****::   :* :*:.:*.:.:.. . .  **: * ***:.*** *: *::

P64604 MLAD_ECOLI         61    GGVVVGRVADITLDPKTYLPRVTLEIEQRYNHI---------------------------      93
V5V921 V5V921_ACIBA       61    SGVTIGRVDSITLDPVTRLATVTFDLDGKLTSFNAEQLKEVQKNALDELRYSSDYTQATP     120
                                .**.:*** .***** * *  **:::: : . :

P64604 MLAD_ECOLI         94    --------------------PDTSSLSIRTSGLLGEQYLALNVGFEDPELGTAILKDGDT     133
V5V921 V5V921_ACIBA      121    AQQKTMEQQLISNMNSITSIDEDAYIMVATNGLLGEKYLKIV-----PGGGLNYLKRGDT     175
                                                    : : : : *.*****:** :       *  *   ** ***

P64604 MLAD_ECOLI        134    IQDTKSAMVLEDLIGQFLYGSKGDDNKN-SGDAPAAAPGNNETTEPVGTTK     183
V5V921 V5V921_ACIBA      176    ISNTQGTMDLEDLISKFITGGGAGKVAAGSSSAEEKAPASTDSSAQPSFVE     226
                                *.:*:.:* *****.:*: *. ...    *..*   **...:::   . .:
```

b

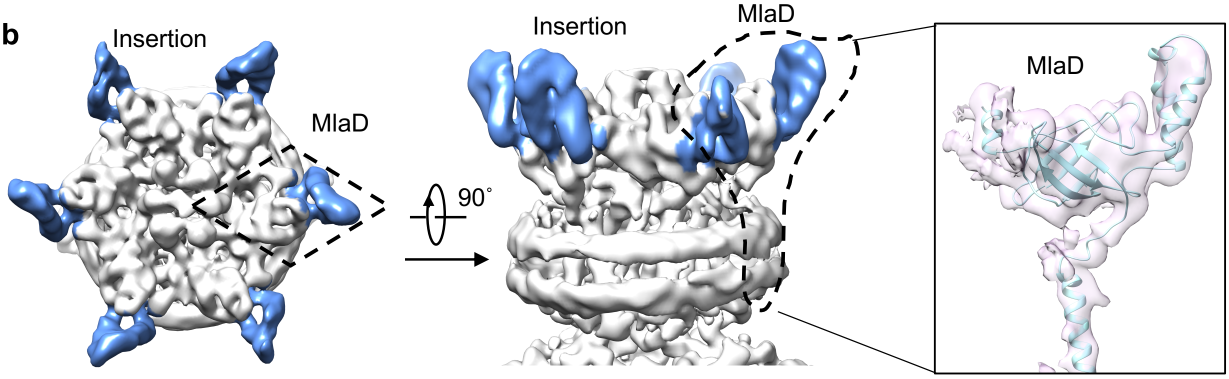

Supplementary Fig. S4 | Overlay of MlaD from *A. baumannii* and *E. coli*. a, Amino acid sequence alignment of MlaDs from *A. baumannii* and *E. coli.* **b,** Density map of MlaFEDB filtered to 6 Å, showing the extra insertion of MlaD from *A. baumannii*.

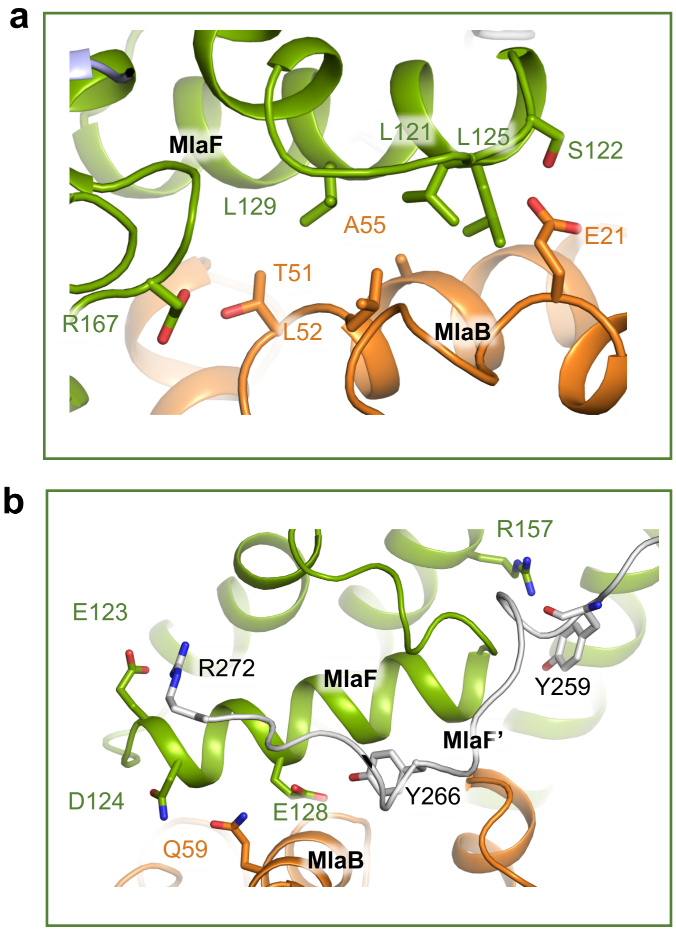

Supplementary Fig. S5 | Interactions of MlaB and MlaF. a, Zoomed-in view of interaction between MlaF and MlaB. **b**, Zoomed-in view of interaction of MlaB, one subunit MlaF’and the other subunit of MlaF.

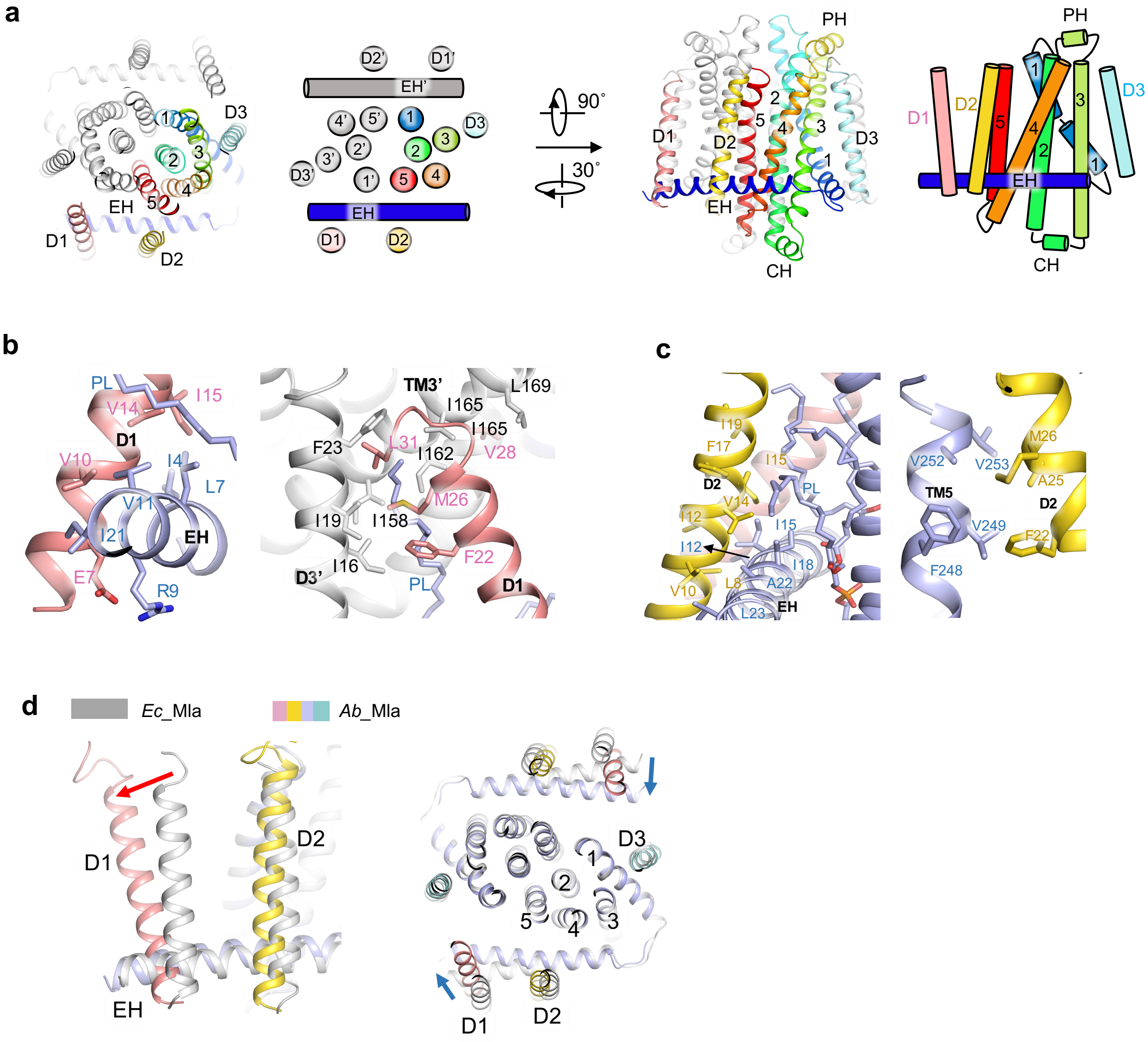

Supplementary Fig. S6 | Interactions of individual subunit in MlaFEDB. a, Periplasm view and side view of the transmembrane helices from MlaE and MlaD. **b**, Zoomed-in views of interaction between transmembrane helix D1 of MlaD and TMs of MlaE . **c**, Zoomed-in views of interaction between transmembrane helix D2 of MlaD and TMs of MlaE . **d**, Zoomed-in views of overlay of TMs from *A. baumannii* and *E. coli.*

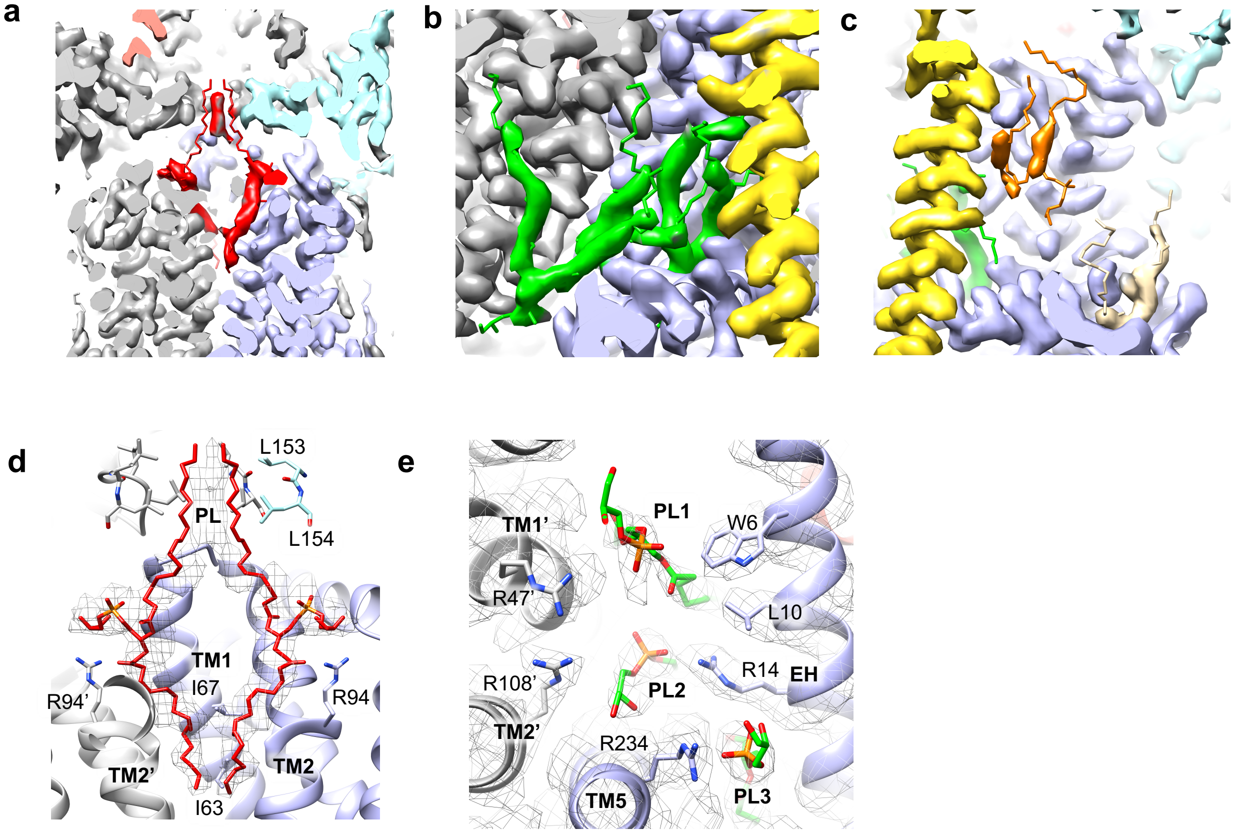

Supplementary Fig. S7 | phospholipid clusters bound in MlaE. a, Zoomed-in view of the phospholipids binding in the central pocket. **b**, Zoomed-in view of the PL1-PL3 binding in the side pocket. **c**, Zoomed-in view of the PL4 and PL5 binding in the side pocket. **e**, Cross-sectional view of the detail interaction between central PLs and MlaE and MlaD. **f**, Periplasmic view of the detail interaction between PL1-PL3 and MlaE.

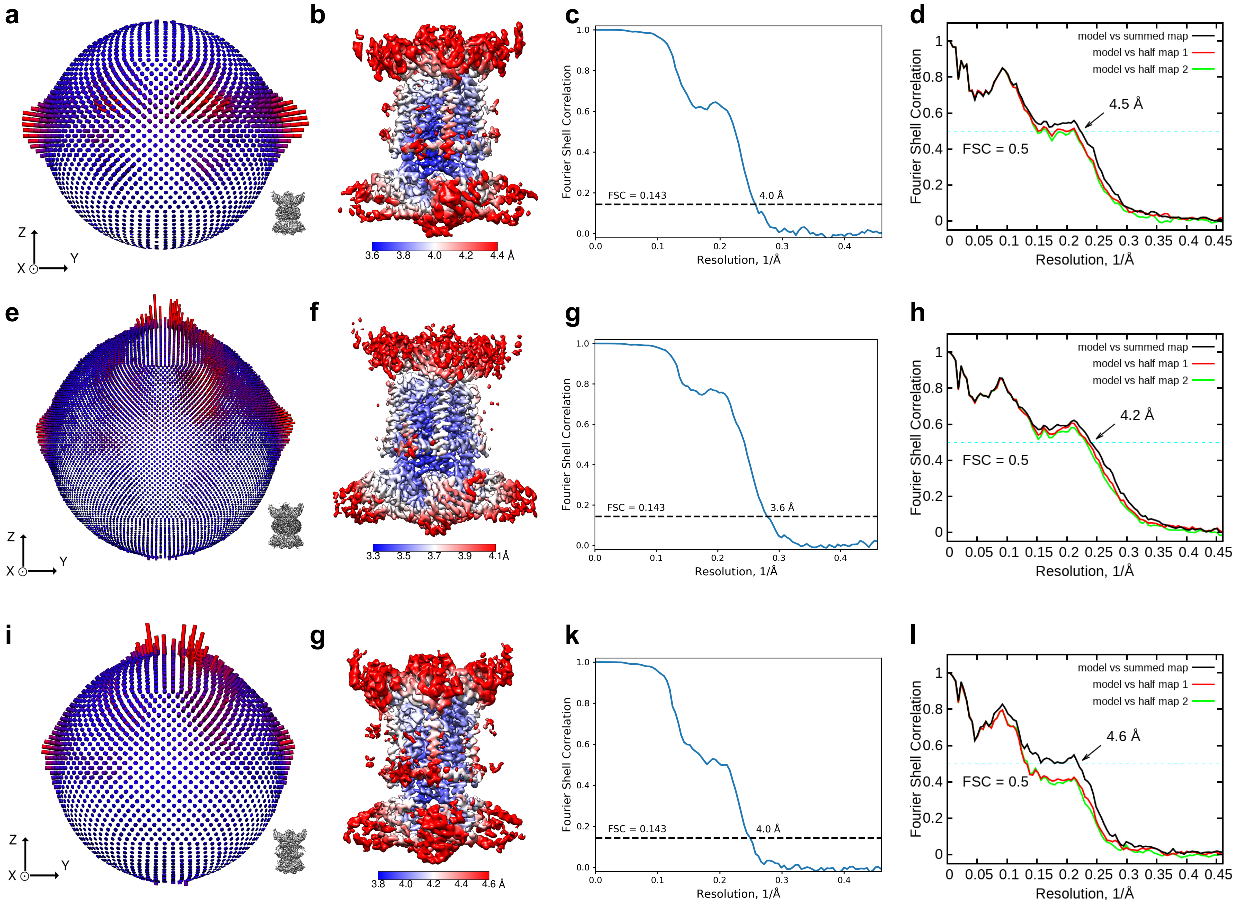

Supplementary Fig. S8 | Single-particle cryo-EM analysis of vanadate-trapped MlaFEDB in nanodiscs. Different subsets of particle images were selected from different classification schemes to produce five high-resolution refined maps. **a-d** cryo-EM analysis of MlaFEDB in V_{trans1} state. **a**, Angular distribution of particle images of MlaFEDB in V_{trans2} state. b, Local resolution of the final cryo-EM map of MlaFEDB in V_{trans1} state. **c**, Gold standard FSC curve for the 3D refinement of MlaFEDB in V_{trans1} state (blue). d, FSC curve of MlaFEDB in V_{trans1}state between the atomic model and the final map with indicated resolution at FSC=0.5 (black); FSC curve between half map 1 (red) or half map 2 (green) and the atomic model refined against half map 1. **e-h** are cryo-EM analysis of MlaFEB in V_{trans2} state, which are same to **a-d** in order. **i-l** are cryo-EM analysis of MlaFEDB in V_{close} state, which are same to **a-d** in order.

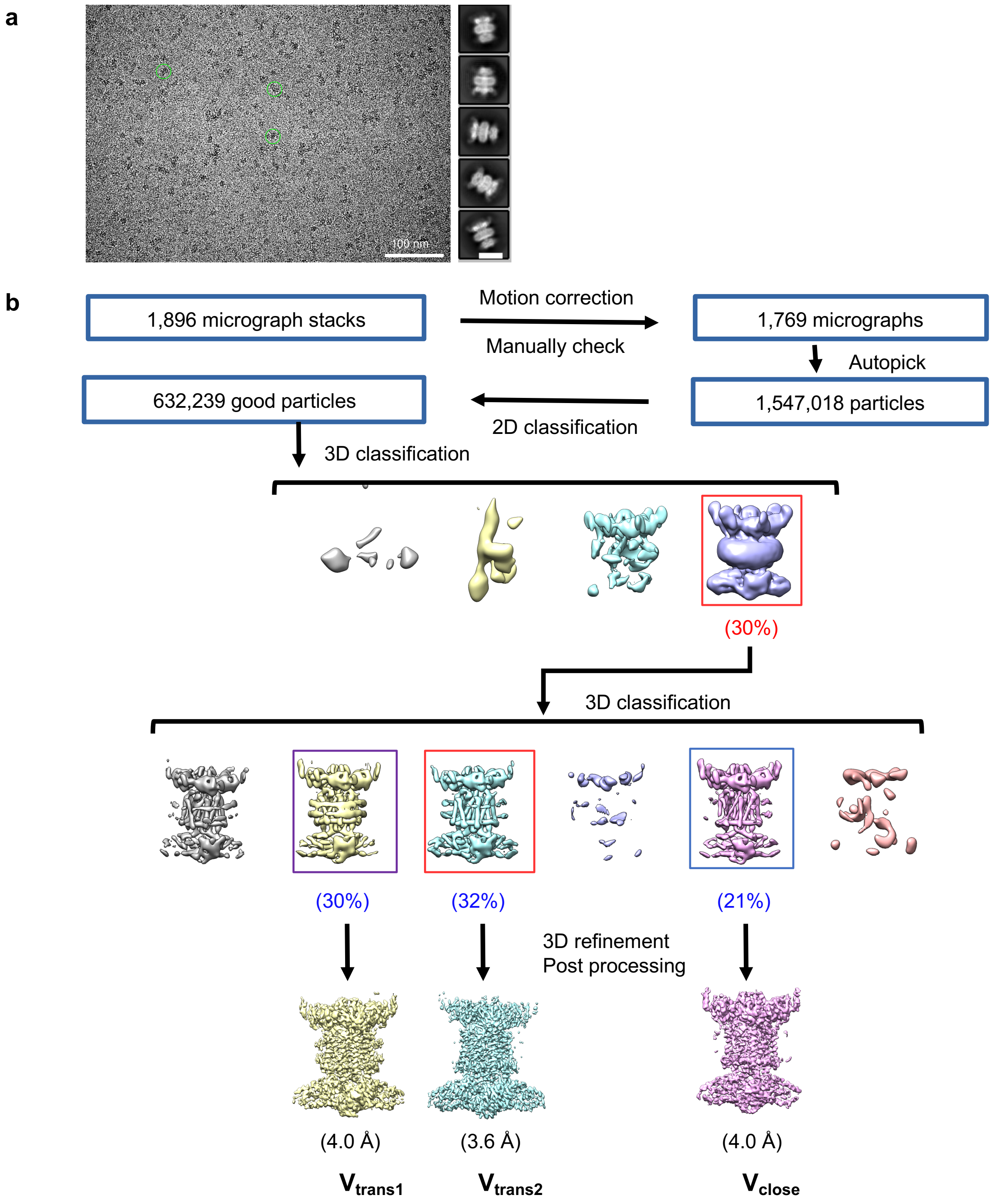

Supplementary Fig. S9 | Image processing for the cryo-EM data of vanadate-trapped MlaFEDB in nanodiscs. a, Representative cryo-EM image and 2D class averages of cryo-EM particle images of vanadate-trapped MlaFEDB in nanodiscs. **b,** Flow chart for cryo-EM data processing. For details, see 'Data processing' in the Methods section.

Supplementary Table S1 | Statistics of the cryo-EM structures presented in this study.

**Data collection**				
EM equipment	Titan Krios (Thermo Fisher Scientific)			
Voltage (kV)	300			
Detector	Gatan K3 Summit			
Energy filter	Gatan GIF Quantum, 20 eV slit			
Pixel size (Å)	1.087			
Electron dose (e-/Å2)	50			
Defocus range (μm)	-1.2 ~ -2.2			
Sample	MlaFEDB complex			
Number of collected micrographs	2,915	1,896		
Number of selected micrographs	2,664	1,769		
**3D Reconstruction**				
Software	Relion 3.0			
	Nucleotide-free	V_{trans1}	V_{trans2}	V_{close}
Number of used particles (Overall)	314,199	48,606	52,789	33,995
Resolution (Å)	3.1	4.0	3.6	4.0
Symmetry	C1			
Map sharpening B-factor (Å2)	120			
**Refinement**				
Software	Phenix			
Cell dimensions				
a=b=c (Å)	217.40			
α=β=γ (˚)	90			
Model composition				
Protein residues	2,374			
Side chains assigned	2,254			
lipid	12			
ADP-vanadate				2
ATP		2	2	
R.m.s deviations				
Bonds length (Å)	0.007	0.006	0.010	0.009
Bonds Angle (˚)	1.170	1.205	1.438	1.697
Ramachandran plot statistics (%)				
Preferred	88.68	89.79	90.21	85.96
Allowed	11.15	9.62	9.45	13.36
Outlier	0.17	0.60	0.34	0.68

SUPPLEMENTARY INFORMATION

Supplementary Movie S1 | Conformational changes of MlaFEDB from nucleotide-free to V_{trans1} state.

Supplementary Movie S2 | Conformational changes of MlaFEDB from nucleotide-free to V_{trans2} state.

Supplementary Movie S3 | Conformational changes of MlaFEDB from nucleotide-free to vanadate-trapped V_{close} state.

Supplementary Methods

MlaFEDB expression and purification. The *mlaFEDCB* operon was amplified from *A. baumannii* genomic DNA by PCR. C-terminal His-tagged MlaFEDCB in pET20b vector was transformed into *E. coli* BL21(DE3) pLyS for expression. Cells were grown at 37°C in Luria broth (LB) medium with 100 μg ml^{-1} Ampicillin and 30 μg ml^{-1} chloramphenicol until the optical density of the culture reached 1.0 at 600 nm. Protein expression was induced with 0.5 mM isopropyl β-D-1-thiogalactopyranoside (IPTG) and cells were grown for 12 h at 16°C. Cells were harvested by centrifugation, flash frozen in liquid nitrogen and stored at -80°C. Frozen cell pellets were re-suspended in lysis buffer containing 25 mM Tris, pH 7.8, 300 mM NaCl and 10% (v/v) glycerol and lysed by microfludizer. Unbroken cells and large debris were removed by centrifugation at 10,000g for 30 min at 4°C. Membranes were pelleted by ultra-centrifugation at 200,000g for 1 hr at 4°C, re-suspended in lysis buffer, and solubilized with 1% (w/v) n-dodecyl-β-D-maltopyranoside (DDM, Anatrace) for 1 hr at 4°C. MlaFEDB were purified over TALON metal affinity resin (Clontech) followed by size-exclusion chromatography on a Superdex 200 column in a buffer containing 25 mM Tris, pH7.8, 150 mM NaCl, 0.05% DDM and 5% glycerol. Protein fractions with the highest homogeneity were collected and concentrated to 6 mg ml^{-1} and stored at -80°C.

Reconstitution of MlaFEDB into nanodisc. 1-palmitoyl-2-oleoyl-sn-glycero-3-phosphoglycerol (POPG) (Avanti Polar Lipids) was solubilized in chloroform, dried under argon gas to form a thin lipid film, and stored under vacuum overnight. The lipid film was hydrated and re-suspended at a concentration of 10 mM POPG. MlaFEDB proteins, MSP1D1 membrane scaffold protein, and POPG were mixed at a molar ratio of 0.5:1:60 in a buffer containing 25 mM Tris, pH 7.8, 150 mM NaCl and 15 mM sodium cholate, and incubated for 1 hr at 4°C. Detergents were removed by incubation with 0.6 mg ml^{-1} Bio-Beads SM2 (Bio-Rad) overnight at 4°C. Nanodisc-embedded MlaFEDB were purified using a Superdex 200 column in a buffer containing 25 mM Tris, pH 7.8, and 150 mM NaCl.

ATPase assay. All ATPase activity assays were performed using ATPase/GTPase activity assay kit(Sigma). Briefly, 0.25 µg of purified MlaFEDB in nanodisc were incubated in a 40 µl reaction volume containing 0-4 mM ATP for 30 min at 37°C. The absorbance at 600 nm was measured. ATPase activities of all samples were determined using the mean value of the samples according to the linear regression of standards.

Electron microscopy sample preparation and data acquisition. To prepare samples for cryo-EM analysis, 3 µl of purified nanodisc-embedded MlaFEDB at a concentration of 5 mg ml^{-1} was applied to glow-discharged holey carbon grids (Quantifoil Au R1.2/1.3). For vanadate trapping, the samples were incubated in a buffer containing 2 mM ATP, 2 mM $MgCl_2$, and 1 mM sodium orthovanadate at room tempture for 30mins before applying the samples to cryo-EM grids. Grids were blotted for 4 s and plunge-frozen in liquid ethane cooled by liquid nitrogen using a Vitrobot MarkIV (ThermoFisher). The prepared grids were transferred to Titan Krios electron microscopy operating at 300 kV equipped with Gatan K3 detector and GIF Quantum energy filter. The movie stacks were automatically collected using AutoEMation[1]with slit width of 20 eV on the energy filter in super-resolution mode at nominal magnification of 81,000×. The defocus range was from -1.5 µm to 2.0 µm. Each stack included 32 frames with an exposure time of 0.08 s for each frame and a total dose rate of about 50 $e^-/Å^2$. The stacks were motion corrected MotionCor2[2] and binned 2-fold, resulting in a pixel size of 1.087 Å / pixel. The dose weighting[3] was operated and the defocus values were estimated by Gctf[4].

Data processing. *A. baumannii* nucleotide-free MlaFEDB particles were automatically picked using Relion 3.0.6 [5-8] from manually selected micrographs. After 2D classification with Relion, good particles were selected and subjected 3D classification using the cryo EM map of the *E. coli* MlaFEDB complex as the initial model with C1 symmetry. Good particles were selected and subjected to further 3D classification, 3D auto-refinement and post-processing. The particle picking and 2D classification of *A. baumannii* vanadate-trapped MlaFEDB sample was similar to nucleotide-free MlaFEDB sample. Good particles selected from 2D classification were 3D classified into six classes with C1 symmetry. Good classes were subjected to 3D classification and 3D auto-refinement and post-processing individually focused on whole structure.The resolution was estimated with the gold-standard Fourier shell correlation 0.143 criterion[9] with high-resolution noise substitution[10]. Refer to Table S1 for details of data collection and processing.

Model building and refinement. Model building of the *A. baumannii* nucleotide-free MlaFEDB was performed by molecular dynamics flexible fitting (MDFF)[11]of the *E. coli* nucleotide-free MlaFEDB complex in the accompany study. A Chainsaw[12] model of the *A. baumannii* nucleotide-free MlaFEDB complex was first obtained using the structure of the *E. coli* nucleotide-free MlaFEDB complex as a template, which was further performed in the cryo-EM map with Phenix[13] and Coot[14] based on the focused-refined cryo-EM maps with aromatic residues as landmarks, most of which were clearly visible in the cryo-EM map. Each residue was manually checked with the chemical properties taken into consideration during model building. Several segments, whose corresponding densities were invisible, were not modeled the sidechain. For different conformations of *A. baumannii* MlaFEDB in the presence of ATP or ADP-VO4, the atomic models were generated by MDFF based on the *A. baumannii* nucleotide-free MlaFEDB model into the corresponding cryo-EM map. These models were further manually refined and checked in Coot. Structural refinement was performed in Phenix with secondary structure and geometry restraints to prevent overfitting. To monitor the potential overfitting, the model was refined as compared to one of the two independent half maps from the gold-standard 3D refinement approach. Statistics associated with data collection, 3D reconstruction and model building were summarized in Table S1.

References for Methods

1. J. Lei, J. Frank, Automated acquisition of cryo-electron micrographs for single particle reconstruction on an FEI Tecnai electron microscope. Journal of structural biology 150, 69-80 (2005).
2. S. Q. Zheng et al., MotionCor2: anisotropic correction of beam-induced motion for improved cryo-electron microscopy. Nature methods 14, 331-332 (2017).
3. T. Grant, N. Grigorieff, Measuring the optimal exposure for single particle cryo-EM using a 2.6 A reconstruction of rotavirus VP6. eLife 4, e06980 (2015).
4. K. Zhang, Gctf: Real-time CTF determination and correction. Journal of structural biology 193, 1-12 (2016).
5. J. Zivanov et al., New tools for automated high-resolution cryo-EM structure determination in RELION-3. eLife 7, (2018).
6. D. Kimanius, B. O. Forsberg, S. H. Scheres, E. Lindahl, Accelerated cryo-EM structure determination with parallelisation using GPUs in RELION-2. eLife 5, (2016).
7. S. H. Scheres, RELION: implementation of a Bayesian approach to cryo-EM structure determination. Journal of structural biology 180, 519-530 (2012).
8. S. H. Scheres, A Bayesian view on cryo-EM structure determination. Journal of molecular biology 415, 406-418 (2012).
9. P. B. Rosenthal, R. Henderson, Optimal determination of particle orientation, absolute hand, and contrast loss in single-particle electron cryomicroscopy. Journal of molecular biology 333, 721-745 (2003).
10. S. Chen et al., High-resolution noise substitution to measure overfitting and validate resolution in 3D structure determination by single particle electron cryomicroscopy. Ultramicroscopy 135, 24-35 (2013).
11. L. G. Trabuco, E. Villa, K. Mitra, J. Frank, K. Schulten, Flexible fitting of atomic structures into electron microscopy maps using molecular dynamics. Structure (London, England : 1993) 16, 673-683 (2008).
12. M. D. Winn et al., Overview of the CCP4 suite and current developments. Acta Crystallogr D Biol Crystallogr 67, 235-242 (2011).

13. P. D. Adams et al., PHENIX: a comprehensive Python-based system for macromolecular structure solution. Acta crystallographica. Section D, Biological crystallography 66, 213-221 (2010).

14. P. Emsley, B. Lohkamp, W. G. Scott, K. Cowtan, Features and development of Coot. Acta crystallographica. Section D, Biological crystallography 66, 486-501 (2010).
